# Getting closer to the clinic

**DOI:** 10.7554/eLife.77180

**Published:** 2022-02-25

**Authors:** Toshiko Tanaka, Luigi Ferrucci

**Affiliations:** 1 Intramural Research Program, National Institute on Aging, National Institutes of Health Baltimore United States

**Keywords:** biomarker, proteomics, epigenetic, prediction, morbidity, aging, Human

## Abstract

Associations between plasma protein levels and DNA methylation patterns can be used to predict the onset of age-related chronic disease.

**Related research article** Gadd DA, Hillary RF, McCartney DL, Zaghlool SB, Stevenson AJ, Cheng Y, Fawns-Ritchie C, Nangle C, Campbell A, Flaig R, Harris SE, Walker RM, Shi L, Tucker-Drob EM, Gieger C, Peters A, Waldenberger M, Graumann J, McRae AF, Deary IJ, Porteous DJ, Hayward C, Visscher PM, Cox SR, Evans KL, McIntosh AM, Suhre K, Marioni RE. 2022. Epigenetic scores for the circulating proteome as tools for disease prediction. *eLife* **11**:e71802. doi: 10.7554/eLife.71802.

Note added [December 5, 2023]

This Insight article has been updated to reflect a correction made to the associated Research Article: in the sixth paragraph, the number of connections between EpiScores and 11 diseases or death has been changed from 137 to 130.

Lengthening life expectancies and decreased mortality rates have led to an unprecedented expansion of the older population. At the same time, chronic diseases – which often affect older individuals – have become more prevalent. Healthcare systems around the world are falling short of this challenge, in part because they remain focused on preventing and curing one disease at a time, even though 80% of clinical patients over 60 have multiple diseases at once.

Even when one specific disease causes most of a person’s symptoms, older patients often have co-existing conditions that affect the course, treatment and prognosis of the main disease. Pressed for time, physicians often ignore underlying illnesses until they begin to seriously affect the patient’s health or start causing frailty. There is no easy solution to this rising crisis, but the emerging field of biomarkers may soon come to the aid of clinicians.

Biomarkers are molecules, genes or characteristics that can be used to detect or predict the onset of a disease. Traditionally, biomarkers have included circulating levels of plasma proteins, lipids and other metabolites. More recently, epigenetic markers – chemical modifications of DNA that affect whether genes are turned on or off, such as addition of methyl groups at specific DNA sites – have shown promise as biomarkers for age-related conditions. Using biomarkers could allow physicians to obtain a molecular map of a patient’s health from a single drop of blood. This would allow clinicians to detect illnesses before they become symptomatic, which is particularly important in the case of serious conditions that could become chronic (Tanaka et al., 2020).

Developing algorithms that extract the relevant information from biomarkers in the blood is perhaps the most promising and potentially powerful line of research in chronic diseases. Until recently, most biomarker studies examined one layer of information (DNA modifications, protein levels or specific metabolites) at a time. However, combining information on DNA methylation with the level of a small number of circulating proteins has been shown to predict the risk of specific chronic diseases as well as global, adverse health outcomes such as having several illnesses at once, and mortality (Lu et al., 2019; Levine et al., 2018; Belsky et al., 2020). Now, in eLife, Riccardo Marioni from the University of Edinburgh and colleagues – including Danni Gadd, Robert Hillary, Daniel McCartney and Shaza Zaghlool as joint first authors – report on how to leverage the associations between DNA methylation and protein levels to predict the onset of disease earlier and more accurately (Gadd et al., 2022).

The team (who are based in the United Kingdom, the United States, Germany, Australia and Qatar) first measured the abundance of 953 proteins in the blood plasma of people in the German KORA cohort (an epidemiological study that ran from 1984 to 2001 in Augsburg and evaluated participants every five years, with an emphasis on major chronic diseases) and the Scottish Lothian Birth Cohort 1936 (the surviving participants of the Scottish Mental Survey 1947 who now live in the Lothian area of Scotland). Gadd et al. then used machine learning to identify clusters of specific DNA methylation sites that could predict the levels of each protein in the plasma. This data was used to assign an epigenetic score or ‘EpiScore’ to each protein. Using this approach, Gadd et al. found that their new algorithm could predict between 1% and 58% of the variation between different people in the plasma levels of 109 proteins.

Next, the team applied the EpiScores of the 109 proteins to data from an independent epidemiological study called Generation Scotland to test whether it was possible to predict the onset of 11 major chronic diseases, as well as death, over a follow-up period of 14 years (Figure 1). This resulted in the identification of 130 connections between EpiScores and 11 diseases or death. Some EpiScores predicted the onset of selected conditions but other were associated with multiple conditions and, perhaps unsurprisingly, the results also suggested a strong correlation between inflammation and age-related chronic disease.

**Figure 1.**
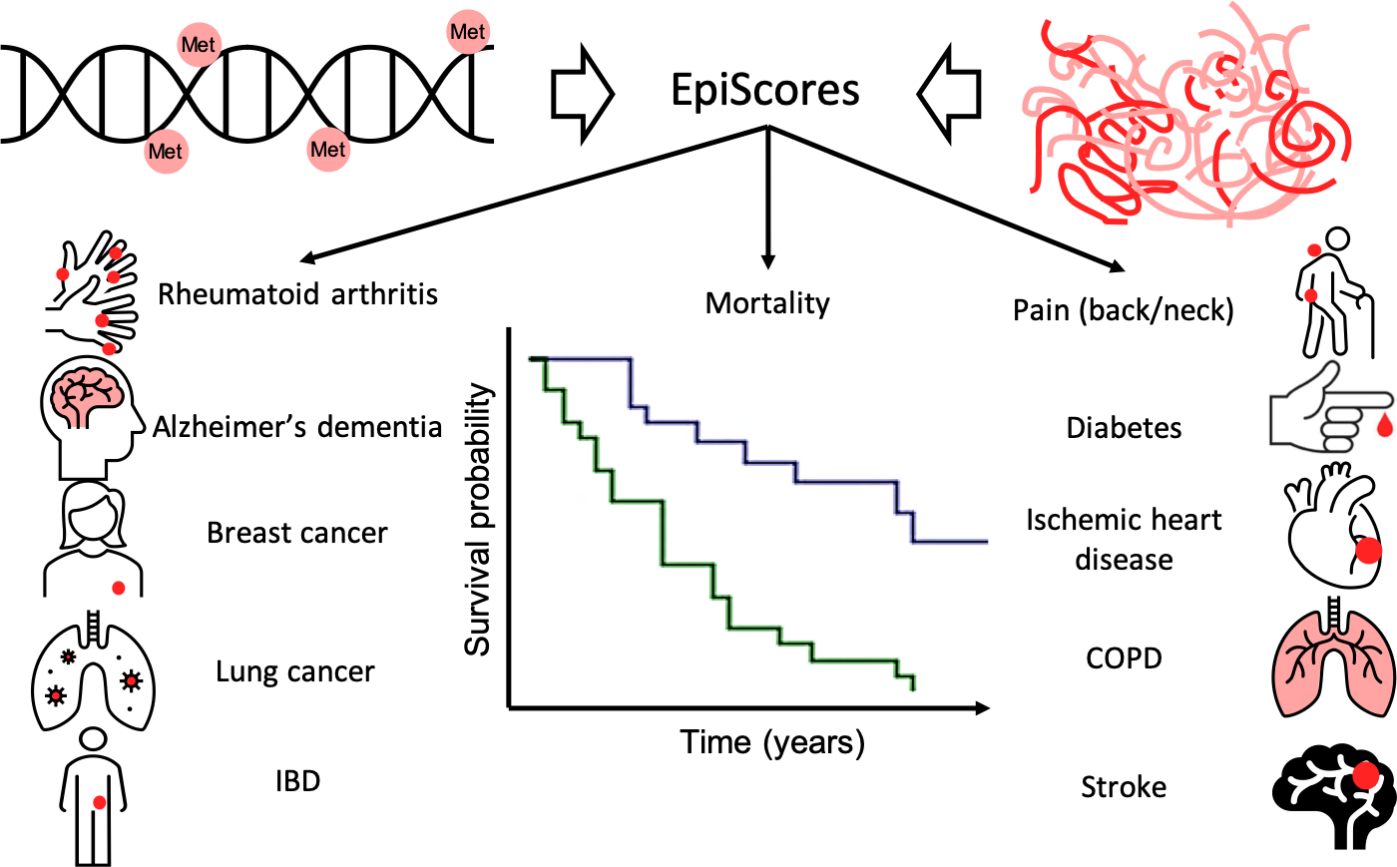
Epigenetic scores of plasma proteins predict onset of major chronic diseases over 14 years. A machine learning approach was used to find associations, called EpiScores, between DNA methylation (top left) and the abundance of 953 plasma proteins (top right). The results identified 109 proteins with EpiScores that explained between 1% and 58% of the variance in their levels. These scores were then applied to an epidemiological study that contains the medical records of 1,537 individuals over the course of 14 years. Gadd et al. found 130 connections between these EpiScores and 11 age-related conditions (represented by icons), and also between the EpiScores and mortality (represented by the survival graph).

One of the notable observations (that has also been reported in previous studies) is that these analyses on EpiScores confirmed known associations between certain proteins and diseases, even when there is only a moderate correlation between the EpiScore and the protein. This suggests that EpiScores are not a mere proxy for plasma protein levels, but may contain different information about disease risk. In the future, it is likely that biomarkers for disease will encompass multiple molecular layers, such as protein levels together with epigenetic markers or metabolite composition.

The findings of Gadd et al. offer a glimpse into a possible future of medicine. One could imagine a busy physician evaluating a 75-year-old patient complaining of sudden back pain. The physician collects a small blood sample and analyzes it using a fast robotized laboratory connected to a powerful computer that can measure molecular biomarkers and assign a ‘health score’. The computer would then provide information about the patient’s risk for potential diseases that the physician can address before they become symptomatic. The systematic use of this technology could increase awareness and understanding of co-existing, but not yet visible, medical problems.

Of course, before this can happen more research is needed. The predictivity of some EpiScores is modest and only adequate for risk prediction. Even in this context, it would be important to understand whether performing early interventions on patients with high scores is cost effective. As always in prevention, there is a trade-off between the stigmata of tagging an individual as ‘high risk’ and how this information can be used to improve health. A study in which information about proteins and DNA methylation is first compared ‘head to head’ in the same large cohort, and then combined, could reveal whether these two biomarkers provide complementary information and increase specificity. Over time, the data collected systematically using this approach and surveillance studies of electronic medical records could help identify common co-morbidities, allowing clinicians to develop more effective strategies for treating patients with complex combinations of diseases.
